# Addressing Needs of Older Adults in Low-Income Independent Living Facilities in Community Health

**DOI:** 10.1093/geroni/igab046.1269

**Published:** 2021-12-17

**Authors:** Nancy Dudley, Lisa Rauch, Toby Adelman, Daryl Canham

**Affiliations:** 1 San Jose State University, Los Altos, California, United States; 2 San Jose State University, San Jose, California, United States

## Abstract

Access to quality care in long-term care settings including independent living facilities is needed for a diverse high-risk aging U.S. population. There is an urgent need to assess and address complex care needs of older adults living longer with chronic conditions and serious illness. However, a system to assess and identify health problems, intervene, and evaluate outcomes is lacking. This session presents learnings from a pilot study developed in collaboration with Nurse Managed Centers at low-income independent living facilities for older adults and undergraduate nursing students in community health practice. We will discuss the adaptation of the Omaha System for provision of care in independent living facilities to address complex care needs. Finally, we will discuss the impact of this project and its potential for healthcare transformation in independent living facilities and transformation of education in undergraduate nursing programs.

